# Practical tools to identify short children born small-for-gestational-age eligible for rhGH treatment according to Italian regulation

**DOI:** 10.1186/s13052-019-0715-x

**Published:** 2019-10-21

**Authors:** Gianluca Tornese, Flavia Pricci, Maria Chiara Pellegrin, Marika Villa, Daniela Rotondi, Elvira Agazio, Egidio Barbi

**Affiliations:** 1 Institute for Maternal and Child Health IRCCS “Burlo Garofolo”, Trieste, Italy; 2 Department Cardiovascular, endocrine-metabolic disease and aging, Istituto Superiore di Sanità, Rome, Italy; 3 University of Trieste, Trieste, Italy

**Keywords:** Growth hormone, Small for gestational age, Italy

## Abstract

Recombinant human growth hormone (rhGH) is an approved and effective treatment for short children born small for gestational age (SGA). Prevalence of children eligible for treatment as SGA is reported to be 1:1800. The latest data from the National Registry of Growth Hormone therapy (RNAOC) showed that the number of children treated with SGA indication is still small (prevalence 0.37/100,000) and these children are significantly less reported than those treated for growth hormone deficiency (GHD), although GHD prevalence is 1:4000–1:10,000. This means that many short children born SGA are still not properly identified, and therefore not treated with rhGH, or misdiagnosed as GHD. This article provides some practical tools for the identification of children eligible for rhGH treatment.

## Background

To born small for gestational age (SGA) is considered the main reason for short stature in 10% of short adults.

Children born SGA should be 2% of the population by definition (− 2 SDS correspond to the 2nd percentile). Actually, some population studies have shown different prevalence: 3.1% in Finland out of 1,390,165 singletons, 5.5% in Sweden out of 3650 healthy full-term children (37–43 gestational weeks) (of which 1.6% SGA only for weight, 2.4% SGA only for length, 1.5% SGA both for weight and length), 3.5% in Japan on 27,228 children (3.4% in term children, 5.5% in preterm children; 1.2% SGA only for weight, 1.5% SGA only for length, 0.8% SGA both in weight and length) [1].

Catch-up growth is more pronounced during the first 6 months and is usually completed in the first 2 years of life (although preterm born SGA catch-up growth is completed also beyond the first 2 years of life, beyond age 6 years and sometimes in adolescence). Previous studies found that 8–12% of SGA children will have a short stature at 2 years of life and these children have a higher risk of short stature later in life [2, 3].

Recombinant human growth hormone (rhGH) is an approved and effective treatment for short children born SGA [4]. Although long-term treatment with rhGH can increase adult height, since SGA children are increasingly recognized as a heterogeneous group in which multiple mechanisms of growth retardation and metabolic disturbances could be causative, it has to be kept in mind – and shared with parents prior to treatment – that the response to rhGH therapy is highly variable and additional studies are needed to identify the responders [5].

SGA children do not need to be deficient in growth hormone (GH) in order to qualify for, or benefit from, rhGH therapy: in fact, the vast majority of SGA children demonstrate GH levels in the normal range, but appear to have low normal circulating IGF-I concentrations. Moreover, SGA children with GH deficiency need higher than replacement dose of rhGH for optimum growth response (0,035 mg/kg/day), probably because of some IGF-I resistance [6]. Assessment of the GH-IGF-I axis may be required if growth velocity is persistently reduced and signs of GH deficiency or hypopituitarism are present.

With the limits of a mechanistic calculus, considering a minimum prevalence of 2% children born SGA for weight and/or length, the hypothetical prevalence of short children born SGA at the age of 2 years would be 0.24% (1:417). Only one Japanese study [1] verified the prevalence of children with short stature born SGA by studying an entire cohort of nearly 30,000 children born during a three-year period and re-evaluated at 3 years of life: the prevalence was found to be 0.06% (1:1800) (notably greater in preterm births < 34 SGA, 0.39%, 1:256). Although considerably lower than the hypothetical one, this prevalence far exceeds the estimated prevalence of GHD (1:4000–1:10,000).

Although rhGH treatment is not mandatory in every child born SGA without catch-up growth, and parents need to be informed about the variability of response to therapy, we believe that in Italy many short children born SGA are still not properly identified and therefore rhGH treatment is not offered as an option.

Aim of this article is to describe the situation of rhGH prescriptions in short children born SGA in Italy and to provide practical tools for pediatricians to identify children eligible for rhGH treatment according to Italian regulation.

## Main text

Treatment with rhGH has been approved in 2009 by the Italian Medicines Agency (AIFA) for the treatment of children with short stature born SGA and is then reimbursed by the Italian National Health System (Servizio Sanitario Nazionale – SSN) according to the Note #39 on the use of drugs.

This treatment was approved in 2001 from the American Food and Drug Administration (FDA), in 2003 from the European European Agency for the Evaluation of Medicinal Products (EMEA) and in 2008 from the Ministry of Labor and of Welfare in Japan; eligibility criteria are slightly different from each other.

**Fig. 1:**
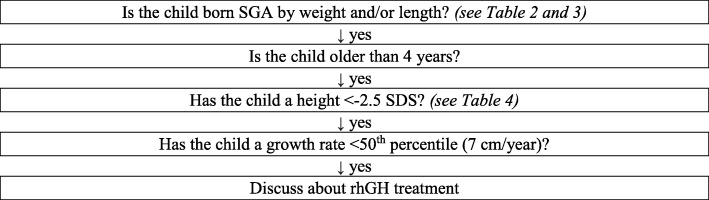
Flow chart to verify the eligibility of a child born SGA for rhGH treatment7

According to the latest version (2014) of the Note #39 of AIFA [7], to access treatment with rhGH in individuals born SGA it is necessary to meet all the following criteria (Fig. 1):

- birth weight ≤ − 2 standard deviations score (SDS) (<3rd percentile) and/or length at birth ≤ − 2 SDS for gestational age according to Bertino charts [8];
- age at the start of GH therapy equal to or greater than 4 years;
- height less than or equal to − 2.5 SDS;
- growth velocity lower than 50th percentile.

In previous regulations, rhGH was provided only for short children born SGA for weight (not for length) and Gagliardi charts had to be used. Bertino charts are the most recent and methodologically more accurate, which differentiate neonatal weights and lengths not only based on sex but also on birth order.

The latest data from the National Registry of Growth Hormone therapy (RNAOC) showed only 311 rhGH prescriptions based on SGA indication versus 3942 based on GHD indication on the overall population of 4584 subjects taking rhGH in childhood in Italy [9]. Moreover, a subanalysis on Registry data, showed that the prevalence of SGA treated patients in 2017 was 0.37/100,000 (1.79/100,000 when considering the 0–15 years population) which is extremely lower than expected, with a stable incidence of new treated patient over the last 7 years (0.42/100,000 per year) (Table 1*)*.

**Table 1:** Prevalence and incidence of rhGH treatment in SGA patients in Italy from 2011 to 2017, according to the National Registry of Growth Hormone therapy data

	Year								
	2011	2012	2013	2014	2015	2016	2017	Mean	±DS
Prevalence									
SGA treated patients (n)	54	68	65	70	108	140	155		
Prevalence (per 100,000 general population/year)	0.37	0.47	0.44	0.28	0.22	0.27	0.37	0.35	0.09
Prevalence (per 100,000 0–15 years population/year)	0.61	0.76	0.72	0.78	1.22	1.60	1.79	1.07	0.47
Incidence									
New SGA treated patients (n)	39	29	30	30	47	49	39		
Incidence (per 100,000 general population/year)	0.37	0.27	0.22	0.28	0.44	0.47	0.38	0.35	0.09
Incidence (per 100,000 0–15 years population/year)	0.44	0.33	0.27	0.34	0.53	0.56	0.45	0.42	0.11

In order not to lose short children born SGA, it would be appropriate for primary care pediatricians to identify their own SGA patients since the first medical encounter and to monitor them over time.

Remarkably, the definition of SGA used in neonatology might sometimes be different from the one reported here (<− 2 SDS): the definition of <10th percentile is broadly used to increase sensitivity for hypoglycemic screening and/or other charts may be used. Therefore, SGA definition needs to be re-evaluated.

Pediatricians can use Bertino charts [8] or, more precisely, directly verify the SDS on the website (http://www.inescharts.com) designed by scientific societies (Italian Society of Pediatric Endocrinology and Diabetology – SIEDP, Italian Society of Neonatology – SIN, Italian Society of Medical Statistics and Clinical Epidemiology – SISMEC); however, to facilitate the identification of SGA children, we propose to use two simple tables (Table 2 for weights and Table 3 for lengths): if the weight or the length of a baby is under the data reported in the Tables, it might be SGA therefore an exact calculation is needed through the aforementioned website to confirm it (data in the Tables are referred to a gestational age of X week + 6 days, which is the highest in the gestational week, in order not to lose data, with the exclusion of 42 weeks + 3 days which is the maximum included in Bertino charts).

**Table 2:** Weight (in grams) that might be ≤ − 2 SDS according to Bertino charts based on gestational age, sex and birth order calculated based on the formula: $\mathrm{SDS}=\frac{{\left(\frac{y}{\mathrm{M}\left(\mathrm{t}\right)}\right)}^{\mathrm{L}\left(\mathrm{t}\right)}-1}{\mathrm{S}\left(\mathrm{t}\right)\times \mathrm{L}\left(\mathrm{t}\right)}$

Gestational age	Firstborn males	Not-firstborn males	Firstborn females	Not-firstborn females
23	379	397	355	373
24	408	429	381	402
25	449	472	418	441
26	501	527	466	492
27	566	597	527	557
28	647	681	602	636
29	745	784	693	732
30	861	906	803	847
31	999	1049	933	983
32	1160	1217	1085	1142
33	1345	1409	1260	1324
34	1552	1625	1457	1529
35	1779	1860	1672	1753
36	2015	2105	1896	1986
37	2244	2344	2114	2213
38	2448	2555	2306	2414
39	2606	2720	2456	2570
40	2711	2830	2555	2673
41	2767	2889	2606	2729
42	2782	2905	2619	2742

**Table 3:** Length (in centimeters) that might be ≤ − 2 SDS according to Bertino charts based on gestational age, sex and birth order calculated based on the formula: $\mathrm{SDS}=\frac{{\left(\frac{y}{\mathrm{M}\left(\mathrm{t}\right)}\right)}^{\mathrm{L}\left(\mathrm{t}\right)}-1}{\mathrm{S}\left(\mathrm{t}\right)\times \mathrm{L}\left(\mathrm{t}\right)}$

Gestational age	Firstborn males	Not-firstborn males	Firstborn females	Not-firstborn females
23	26.3	26.6	25.8	26.1
24	27.4	27.7	26.9	27.2
25	28.6	28.9	28.1	28.3
26	29.8	30.1	29.3	29.5
27	31.1	31.4	30.5	30.8
28	32.4	32.7	31.8	32.1
29	33.7	34.1	33.1	33.4
30	35.1	35.5	34.5	34.8
31	36.5	36.9	35.9	36.2
32	38.0	38.4	37.3	37.7
33	39.5	39.8	38.7	39.1
34	40.9	41.3	40.1	40.5
35	42.3	42.7	41.5	41.9
36	43.6	44.0	42.8	43.2
37	44.8	45.2	44.0	44.4
38	45.8	46.3	45.0	45.4
39	46.7	47.1	45.8	46.3
40	47.3	47.8	46.4	46.9
41	47.8	48.2	46.9	47.4
42	48.0	48.4	47.1	47.5

If the child born SGA has a height lower than − 2.5 SDS at 4 years and growth rate is <50th percentile, there is an indication to start treatment with rhGH and the child must be sent to a pediatric endocrinology center (rhGH prescription is subject to a therapeutic plan, signed by specialized centers which are identified by Regions and Autonomous Provinces). To calculate height SDS, pediatricians can use the Growth Calculator distributed by SIEDP on the website http://www.weboriented.it/gh4/; however, to facilitate the identification of short children we propose to use Table 4. For practical purposes, it may be useful to remind that a growth rate of 7 cm in the previous year is equal to the 50th percentile according to Tanner charts.

**Table 4:** Height (in centimeters) equal to −2.5 SDS at 4 years of age (Cacciari charts [10] for Italian children or WHO charts [11] for other backgrounds)

	Males	Females
Cacciari charts	91.97	91.55
WHO < 5 years charts	92.85	92

## Conclusions

In conclusion, data suggest that SGA children with short stature are under recognized and frequently miss an opportunity of treatment in Italy. We strongly encourage the use of these simple and time-sparing tables for the identification and appropriate care of SGA children.

## Data Availability

Data sharing is not applicable to this article as no datasets were generated during the current study.

## Abbreviations

GHD: Growth hormone deficiency
rhGH: recombinant human growth hormone
RNAOC: National Registry of Growth Hormone therapy
SGA: Small for gestational age

## References

[1] Fujita K, Nagasaka M, Iwatani S (2016). Prevalence of small for gestational age (SGA) and short stature in children born SGA who qualify for growth hormone treatment at 3 years of age: population-based study. Pediatr Int.

[2] Albertsson-Wikland K, Karlberg J (1997). Postnatal growth of children born small for gestational age. Acta Paediatr Suppl.

[3] Leger J, Limoni C, Czernichow P (1997). Prediction of the outcome of growth at 2 years of age in neonates with intra-uterine growth retardation. Early Hum Dev.

[4] Lee PA, Chernausek SD, Hokken-Koelega AC, Czernichow P (2003). International Small for Gestational Age Advisory Board. International Small for Gestational Age Advisory Board consensus development conference statement: management of short children born small for gestational age, April 24–October 1, 2001. Pediatrics.

[5] Maiorana A, Cianfarani S (2009). Impact of TH therapy on adult height of children born small for gestational age. Pediatrics.

[6] de Zegher F, Francois I, van Helvoirt M (1998). Growth hormone treatment of short children born small for gestational age. Trends Endocrinol Metab.

[7] Italia. Determinazione dell’Agenzia Italiana del Farmaco 19 giugno 2014. Modifica alla Nota AIFA 39. Gazzetta Ufficiale. Serie Generale n. 154 del 5 luglio 2014.

[8] Bertino E, Spada E, Occhi L (2010). Neonatal anthropometric charts: the Italian neonatal study compared with other European studies. J Pediatr Gastroenterol Nutr.

[9] Pricci F, Villa M, Maccari F (2018). The Italian registry of GH treatment: electronic clinical report form (e-CRF) and web-based platform for the national database of GH prescriptions. J Endocrinol Investig.

[10] Cacciari E, Milani S, Balsamo A (2006). Italian cross-sectional growth charts for height, weight and BMI (2 to 20 yr). J Endocrinol Investig.

[11] WHO Multicentre Growth Reference Study Group (2006). WHO child growth standards based on length/height, weight and age. Acta Paediatr.

